# Factors for tumor progression in patients with skull base chordoma

**DOI:** 10.1002/cam4.834

**Published:** 2016-08-21

**Authors:** Liang Wang, Kaibing Tian, Ke Wang, Junpeng Ma, Xiaojuan Ru, Jiang Du, Guijun Jia, Liwei Zhang, Zhen Wu, Junting Zhang

**Affiliations:** ^1^ Department of NeurosurgeryBeijing Tiantan HospitalCapital Medical UniversityTiantan Xili 6BeijingDongcheng Distract100050China; ^2^ China National Clinical Research Center for Neurological DiseasesTiantan Xili 6BeijingDongcheng Distract100050China; ^3^ Department of NeuroepidemiologyBeijing Neurosurgical InstituteTiantan Xili 6BeijingDongcheng Distract100050China; ^4^ Department of NeuropathologyBeijing Neurosurgical InstituteTiantan Xili 6BeijingDongcheng Distract100050China

**Keywords:** Chordoma, nomogram, prognosis, progression‐free survival, skull base

## Abstract

Skull base chordoma is a rare and fatal disease, recurrence of which is inevitable, albeit variable. We aimed to investigate the clinicopathologic features of disease progression, identify prognostic factors, and construct a nomogram for predicting progression in individual patients. Data of 229 patients with skull base chordoma treated by one institution between 2005 and 2014 were retrieved and grouped as primary and recurrent. Kaplan–Meier survival of progression was estimated, taking competing risks into account. Multivariable Cox regression was used to investigate survival predictors. The primary group consisted by 183 cases, gained more benefits on 5‐year progression‐free survival (PFS) (51%) and mean PFS time (66.9 months) than the recurrent group (46 cases), in which 5‐year postrecurrent PFS was 14%, and mean postrecurrent PFS time was 29.5 months. In the primary group, visual deficits, pathological subtypes, extent of bone invasion, preoperative Karnofsky performance scale (KPS) score, and variation in perioperative KPS were identified as independent predictors of PFS. A nomogram to predict 3‐year and 5‐year PFS consisted of these factors, was well calibrated and had good discriminative ability (adjusted Harrell C statistic, 0.68). In the recurrent group, marginal resection (*P *=* *0.018) and adjuvant radiotherapy (*P *=* *0.043) were verified as protective factors associated with postrecurrent PFS. Factors for tumor progression demonstrated some differences between primary and recurrent cases. The nomogram appears useful for risk stratification of tumor progression in primary cases. Further studies will be necessary to identify the rapid‐growth histopathological subtype as an independent predictor of rapid progression.

## Introduction

Chordoma is a rare tumor, which is considered to arise from transformed remnants of notochord with an annual incidence of approximately one case per million 1, 2. As a low‐grade but locally invasive malignant tumor, it has a predilection for the axial skeleton, with the most common sites being the sacrum, skull base, and mobile spine 2, 3. Histopathologically, it is generally classified into three subtypes: conventional, chondroid, and dedifferentiated, while the histopathologic criteria for the dedifferentiated variant is still not clear 4, 5. The features of spreading along critical bony and neural structures, insidious disease course, and radio‐resistance make its clinical management difficult, especially for skull base chordomas, mostly leading to depressing prognosis 1, 3.

For such a disease with low incidence and long disease course, it is difficult to conduct studies focused on the prognostic factors associated with clinical outcome. Limited statistical power and inconsistent results were gained by either systematic studies 6, 7 or comprehensive analyses based on some databases such as Surveillance Epidemiology and End Results (SEER) 2, 8. In addition, the difficulty to supply sufficient sample size, when it was necessary to evaluate the primary and recurrent lesions separately, should also be considered 9, 10.

At skull base center of Beijing Tiantan Hospital, we provided medical care to more than 250 patients with skull base chordomas for the last 10 years. The consistency of treatment philosophy regarding this disease: aggressive surgical resection whenever possible, with radiotherapy, referring mainly to Gamma knife, given as an adjuvant treatment for primary cases with obvious remnants postoperatively or as a savage treatment choice for recurrent cases, ensures the inherent homogeneity and comparability in our cohort.

The current urgency to identify characteristics for reliable risk stratification of tumor progression, which is also critical for treatment choice, actuated a series of retrospective studies by us. The aim of this study was to investigate the clinicopathologic feature of disease progression, to identify the predictors about progression‐free survival in primary and recurrent cases, and to establish an effective prognostic nomogram on tumor progression in patients with primary lesions.

## Materials and Methods

### Patient population

**Figure 1:**
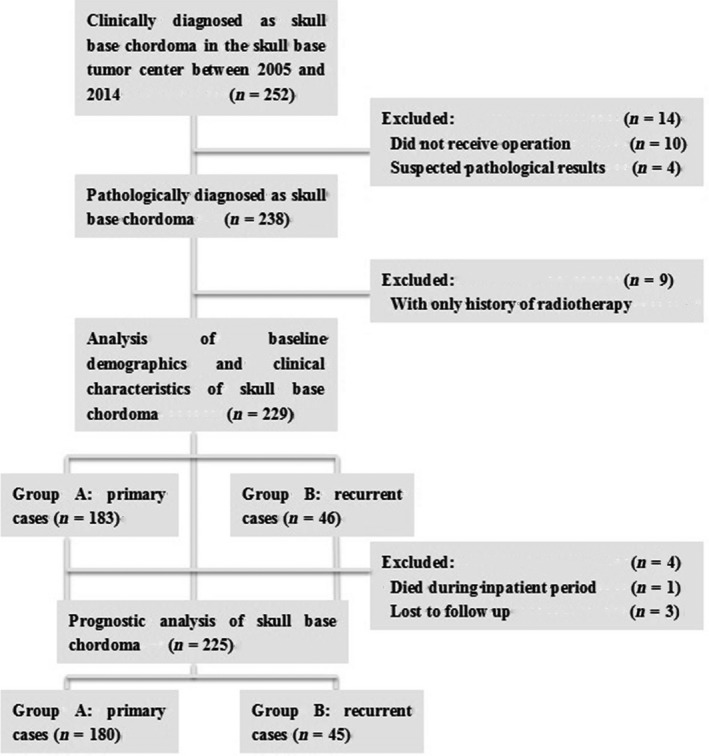
Study flow diagram.

An independent cohort of consecutive patients diagnosed and treated for a skull base chordoma from February 2005 to December 2014 at Skull Base Wards of Beijing Tiantan Hospital, Capital Medical University was analyzed in this retrospective study. With the permission from the Ethics Committees of the same academic medical center, clinicopathologic data were retrieved from a prospectively maintained database. For the patients who received repeated treatments in our center, their first surgery performed by us was set as the cutoff point of enrollment in this study. Patients without any forms of tumor resection, with uncertain pathological results, and with only radiotherapy history before enrollment were excluded from this study. Patients who had been lost to follow‐up were further excluded from the prognostic analysis, as shown in Figure 1.

All patients were recommended to be followed‐up on an outpatient basis at 3‐month intervals for the first follow‐up evaluation, then at 6‐month interval for the second time, and annually for life thereafter. The last update of follow‐up was performed via telephone interviews by two trained researchers in April, 2015 (K.T., K.W.).

### Assay methods

Baseline information retrieved comprised age, sex, diameters, duration of initial symptoms, treatment history, operation time, blood lost, surgical complications, inpatient stays, perioperative Karnofsky performance scale (KPS) scores, which were measured at the first and last day of inpatient care, and updated status from follow‐up procedures.

For the MR imaging features, tumor volume was approximatively calculated by the cubature formula: V=(D1×D2×D3)π/6, in which D1, D2, and D3 represent the longest diameter measurement in three dimensions (sagittal, coronal, and axial), respectively. The evaluations of tumor location and extent of bone invasion were conducted independently by two researchers (L.W., K.T.) according to the classification criterions proposed by our study team. Based on MR images, the definition of bone invasion classification included: (1) Endophytic type, that can invade the bones through every directions, with the transformation of clivus to be like a “bubble” or a “dumbbell”; (2) Intrinsic type, which are relatively rare, represents those inner bony lesions with neither extraosseous nor intraosseous extension trends at the moment; (3) Exophytic type, which had limited bone invasiveness, showing a “bulge‐like” image in the retroclival region.

Regarding the clinical information, initial symptoms were classified as headache and neck pain, diplopia, visual deficits, including hypopsia and hemianopia, cavernous sinus symptoms (composed by proptosis, ophthalmoplegia, ptosis, or trigeminal sensory loss), and others. Surgeries were performed through anterior midline approaches, which consist of microscopic and endoscopic endonasal approaches, or lateral open approaches mainly by two senior physicians (J.Z., Z.W.). A uniform residual tumor classification was utilized on the basis of immediate postoperative MRIs: marginal resection (MR) was defined as greater than 90% excision, and intralesional resection (IR) was defined when less than 90% was resected 11. Whether the skull base dura had been broken through by the lesion was identified on the basis of intraoperative findings.

Radiation therapy, referring mainly to Gamma knife, was recommended to the patients who had obvious and active remnants during their follow‐up evaluations, and those who had locally advanced lesions not amenable to surgery. Adjuvant radiotherapy was defined as radiotherapy within 6 months postoperatively in this study. The information of radiotherapy was gained from the follow‐up system and telephone interviews.

Every specimen was diagnosed as conventional, chondroid, or dedifferentiated subtype according to its histological appearance and results of immunohistochemical staining, including S‐100, CK8/18, EMA, vimentin, brachyury, and Ki‐67 by two isolated pathologists. According to reports 5, 10, 12, 13, 14, 15 and our findings 16, a rapid‐growth subgroup was distinguished from conventional chordoma when it met one of the criteria as following: (1) necrosis and hemorrhage were present; (2) ≥3 mitotic figures were counted in 10 high‐power fields; and (3) Ki‐67 ≥ 6% was identified when available. The results were double‐checked by one pathologist (J.D.), who independently reviewed all slides.

### Study design

The enrolled patients were classified into primary group (Group A) whose lesions were newly diagnosed, and recurrent group (Group B), if they had a history of surgery with or without radiotherapy. The diagnosis of tumor progression, including local recurrence and regrowth, was made by comprehensive considerations of patients' progressive symptoms and detectible radiological changes during follow‐up. The end point of primary group was progression‐free survival (PFS), defined as the time from the start of follow‐up to the event of tumor progression. The end point of recurrent group was postrecurrent PFS (pr‐PFS), defined as the period from the beginning of enrolled follow‐up to tumor progression once again. Patients with no events were censored at the date of their last follow‐up.

### Statistical analysis

Patients' characteristics (demographic, clinicopathologic, and outcomes) were described either by mean (median, interquartile range) for quantitative data, or by counts and percentages for qualitative data. We compared these baseline characteristics between two groups using Pearson's chi‐squared test for categorical variables and the Wilcoxon Mann–Whitney test for continuous variables.

Regarding the comparative survival analysis between the two groups, PFS curves were first estimated by the Kaplan–Meier method, and the two‐sided log‐rank test was performed. Secondly, a bootstrapped Cox proportional hazards model was used to determine significant contributors to differences in PFS and pr‐PFS, respectively. The proportional hazards assumption was checked graphically using log‐log survival plots. Variables were included in the multivariable analysis only if found to be associated with survival (*P* < 0.10) on univariable analysis. Hazard ratios (HRs) and CIs were estimated with Cox proportional hazards regression models to assess the survival outcomes adjusted for baseline demographic and clinicopathologic factors. Moreover, a subset Kaplan–Meier analysis was performed to compare the initial PFS and pr‐PFS in recurrent group.

A nomogram was formulated based on the results of multivariate analysis. A final model selection was performed by a backward stepdown selection process 17. Nomogram performance was assessed by calibration plot as an indicator of internal calibration and by the Harrell C statistic as a measure of discriminative ability. The Harrell C statistic corresponds to the area under the receiver operating characteristic curve; values 0.5 and 1, respectively, indicate lack of discriminative ability and perfect discriminative ability.

*P *< 0.05 was considered statistically significant. All statistical calculations were performed independently by two researchers (L.W., X.R.) using IBM SPSS version 19.0 (IBM, Inc.) and R software (http://www.r-project.org/).

## Results

A total of 229 patients were included, 183 in Group A and 46 in Group B, as provided in Figure 1. Characteristics of the patients are summarized in Table 1. Regarding the baseline parameters, there was no difference between two groups in sex, age, constitution ratios of initial symptoms and surgical approaches, degrees of bone and dura invasion, blood loss, inpatient days, and ratio of surgical complications. Median tumor volume, maximal diameter, and the proportion of extensive type (Type E) in primary group were significantly smaller compared with the recurrent lesions. Correspondingly, patients with recurrent chordomas were less likely to gain marginal resection (32.6% vs. 74.9%, *P *<* *.001). There was no significant difference between Group A and B in the ratio of adjuvant radiotherapy either (20.8% vs. 17.4%, *P *=* *0.777). Twenty‐eight cases in Group A received Gamma knife as their adjuvant radiotherapy (73.7%), and six cases in Group B chose Gamma knife for the adjuvant radiotherapy (75%). As a pathological result, the rapid‐growth subtype accounted for 8.2% of primary lesions and 30.4% of recurrent ones (*P *<* *0.001). And the patients with primary lesions gained obviously better perioperative KPS scores, which meant they kept in better functional status than the recurrent patients perioperatively.

**Table 1:** Baseline demographics and clinical characteristics for patients

Characteristic	Group A		Group B		*P* value
	No.	%	No.	%	
Male	109	59.6	27	58.7	0.915
Age					0.249
Median	40		40.5		
IQR	29–51		31.4–49.6		
Diameter_max_ (mm)					0.001
Median	40.8		46.9		
IQR	31.4–50.2		40.8–53.1		
Volume (mL)					0.002
Median	20.2		33.0		
IQR	6.7–33.8		19.6–46.5		
Initial symptom					0.610
Headache	65	35.5	12	26.1	
Diplopia	52	28.4	13	28.3	
Visual symptoms	20	10.9	5	10.9	
Cavernous sinus symptoms	7	3.8	3	8.7	
Others	39	21.4	13	26	
Duration of initial symptom (m)					0.003
Median	7		3.5		
IQR	0–17.8		1.25–5.8		
Location classification					0.020
SC	111	60.7	25	54.3	
OC	31	16.9	9	19.6	
SP	19	10.4	3	6.5	
PO	13	7.1	2	4.3	
ES	4	2.2	0	0.0	
E	5	2.7	7	15.2	
Bone invasion classification					0.120
Endophytic	143	78.1	42	91.3	
Intrinsic	6	3.3	1	2.2	
Exophytic	34	18.6	3	6.5	
Dura broken	69	37.7	20	43.5	0.473
Approaches					0.642
Midline anterior	38	20.8	11	23.9	
Lateral open	145	79.2	35	76.1	
Marginal resection	137	74.9	15	32.6	<0.001
Operation time (h)					<0.001
Median	6		7.5		
IQR	4.6–7.4		6.3–8.8		
Blood loss (mL)					0.480
Median	700		600		
IQR	450–950		100–1100		
Pathological classification					<0.001
Conventional	105	57.4	17	37	
Rapid‐growth subtype	15	8.2	14	30.4	
Chondroid	63	34.4	15	32.6	
Inpatient days (day)					0.410
Median	19		21		
IQR	13.5–24.5		17.4–24.6		
Preoperative KPS score					<0.001
Median	80		70		
IQR	70–90		60–80		
Postoperative KPS score					<0.001
Median	80		70		
IQR	70–90		65–75		
Perioperative KPS variation					0.563
Stable	109	59.6	26	56.5	
Ascent	46	25.1	10	21.7	
Descent	28	15.3	10	21.7	
Surgical complications	35	19.1	8	17.4	0.788
Surgical mortality	0	0	1	2.2	0.046
Adjuvant radiotherapy	38	20.8	8	17.4	0.389

**Figure 2:**
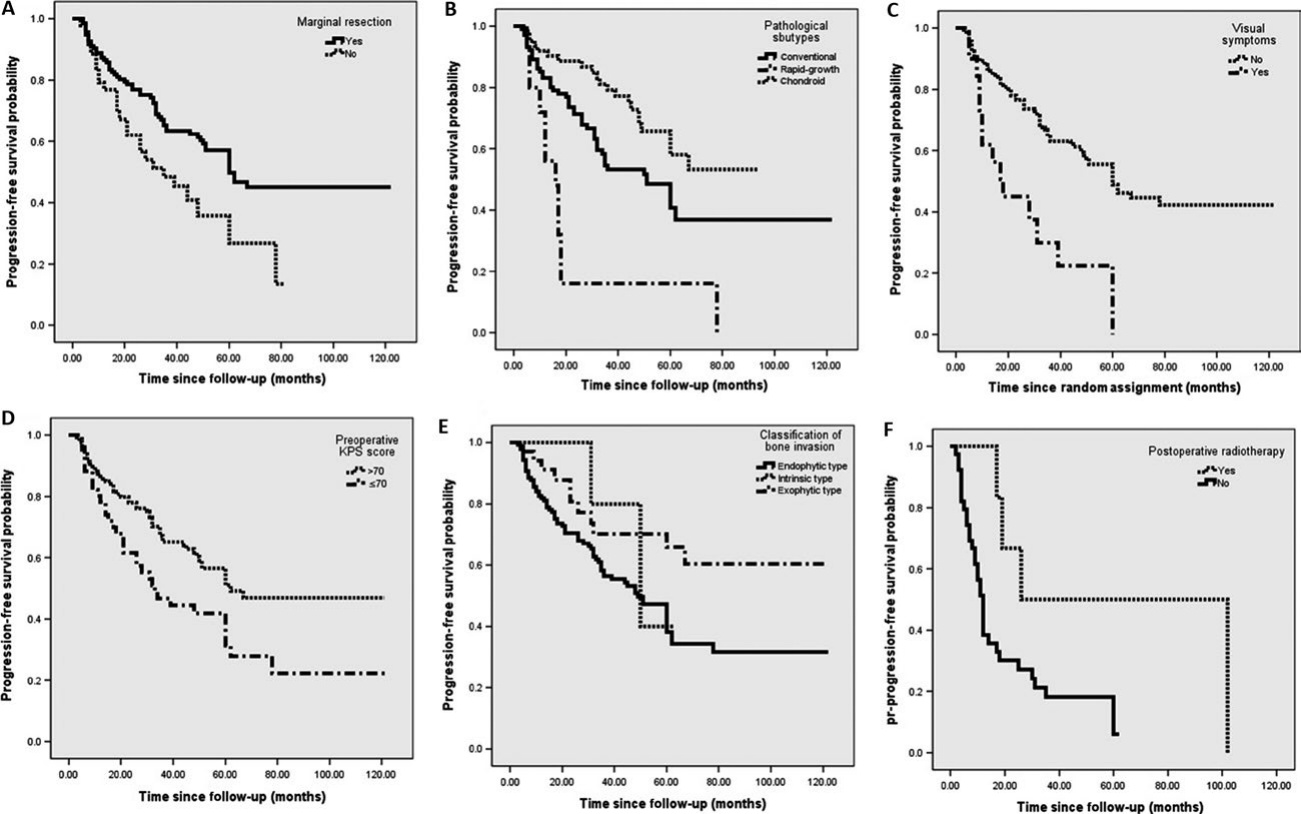
Kaplan–Meier curves of factors for progression‐free survival in primary group, including (A) extent of tumor resection; (B) pathological subtypes; (C) visual symptom; (D) preoperative KPS score; (E) extent of bone invasion, and another factor, (F) adjuvant radiotherapy for pr‐PFS in recurrent group. KPS, Karnofsky performance scale; PFS, progression‐free survival.

IQR, interquartile range; KPS, Karnofsky performance scale; SC, OC, SP, PO, ES, E *cf*.: Figure 2.

Regarding the follow‐up results, the mean follow‐up period was 43.7 months (median: 41 months; range: 4–127 months). Tumor progression was detected in 79 patients of Group A and 34 patients of Group B. As for survival rates, patients with primary lesions underwent significantly longer period without tumor progression (mean PRF time, 66.9 months; 95% CI: 58.9–74.8 months) compared to patients with recurrent lesions (mean pr‐PFS time, 29.5 months; 95% CI: 19.1–40.0 months; *P *<* *.001). The PFS rates of 3‐year and 5‐year were 61% and 51% in the former arm, and 3‐year and 5‐year pr‐PFS were only 23% and 14% in the latter one.

Among the cases with tumor progression, 37 patients (46.8%) in Group A received 49 reoperations. The ratio of reoperation was insignificantly higher compared with Group B, in which 11 patients (32.4%) received cytoreductive surgeries once again (*P *=* *0.111). It was worthy to note that eight patients in Group B did not have radiotherapy in their treatment courses. They chose operation and reoperation as their primary and secondary treatment choices. Their mean initial PFS was 63.8 months, which was significantly longer than their pr‐PFS time (mean: 12.8 months, *P *<* *0.001).

Table 2 depicts PFS estimates of the two arms. According to the univariable analysis, when Group A was concerned, tumor volume ≥20 mL, visual disturbance as the initial symptom, more aggressive bone invasion, anterior midline approach, intralesional resection, preoperative KPS score ≤70, descent trend of perioperative KPS score, and rapid‐growth subtype were significantly associated with earlier tumor progression, as shown in Figure 2A–E. When it came to multivariable analysis, the poorer prognosis categories were visual symptoms and rapid‐growth subtype, while the exophytic type of bone invasion and chondroid subtype of histopathology were confirmed as independent protective factors.

**Table 2:** Univariable and multivariable analyses of factors associated with progression‐free survival

Variable	Group A						Group B					
	Univariable analysis			Multivariable analysis			Univariable analysis			Multivariable analysis		
	HR	95% CI	*P* value	HR	95% CI	*P* value	HR	95% CI	*P* value	HR	95% CI	*P* value
Sex (F vs. M)	0.813	0.527–1.257	0.352				1.083	0.553–2.119	0.816			
Age (≤30 vs.>30)	1.365	0.851–2.190	0.197				1.299	0.567–2.975	0.536			
Volume(mL) (≥20 vs.<20)	0.660	0.428–1.017	0.060			0.316	0.893	0.402–1.985	0.782			
Cavernous sinus symptoms (yes vs. no)	0.892	0.281–2.836	0.847				0.695	0.207–2.337	0.557			
Visual symptoms (yes vs. no)	0.388	0.188–0.606	<0.001	0.402	0.221–0.731	0.003	0.163	0.058–0.461	0.001	0.163	0.058–0.461	0.001
Duration of initial symptom(month) (≤12 vs.>12)	0.882	0.553–1.407	0.598									
History of treatment (m) (≤36 vs.>36)	—	—	—				0.564	0.25–1.116	0.100			0.527
Radiotherapy history (yes vs. no)	—	—	—				0.965	0.486–1.915	0.918			
Location1 (lateral vs. midline)	1.557	0.821–2.953	0.175				1.662	0.503–5.497	0.405			
Location2 (upper vs. lower)	1.362	0.832–2.228	0.219				0.704	0.300–1.649	0.419			
Exophytic growth (yes vs. no)	2.093	1.106–3.961	0.023	1.905	0.995–3.647	0.052	0.326	0.094–1.129	0.077			0.072
Approaches (midline vs. lateral)	0.490	0.305–0.787	0.003			0.313	0.883	0.414–1.885	0.748			
Marginal resection (yes vs. no)	1.832	1.148–2.923	0.011			0.318	2.510	1.174–5.365	0.018			0.172
Complications (yes vs. no)	0.930	0.553–1.566	0.786				0.582	0.241–1.405	0.229			
Preoperative KPS (>70 vs. ≤70)	1.767	1.142–2.735	0.011			0.116	0.995	0.489–2.024	0.988			
Descent perioperative KPS (yes vs. no)	0.627	0.367–1.069	0.086			0.113	0.635	0.274–1.469	0.288			
Adjuvant radiotherapy (no vs. yes)	0.922	0.510–1.667	0.789				0.291	0.088–0.963	0.043			0.094
Pathological subtypes												
Conventional vs. rapid‐growth	3.249	1.170–7.712	<0.001	1.841	0.849–3.992	0.002	1.723	0.804–3.698	0.162	1.841	0.849–3.992	0.122
Conventional vs. chondroid	0.544	0.328–0.904	0.019	0.459	0.189–1.117	0.011	0.441	0.182–1.066	0.069	0.459	0.189–1.117	0.086

HR, hazard ratio.

In the recurrent group, there were some factors that significantly influenced their pr‐PFS time, including adjuvant radiotherapy (Fig. 2F), marginal resection, visual symptoms, and chondroid chordomas, as listed in Table 2. The low sample size in this group, however, made the multivariable analysis of so many factors associated with tumor progression unreliable.

**Figure 3:**
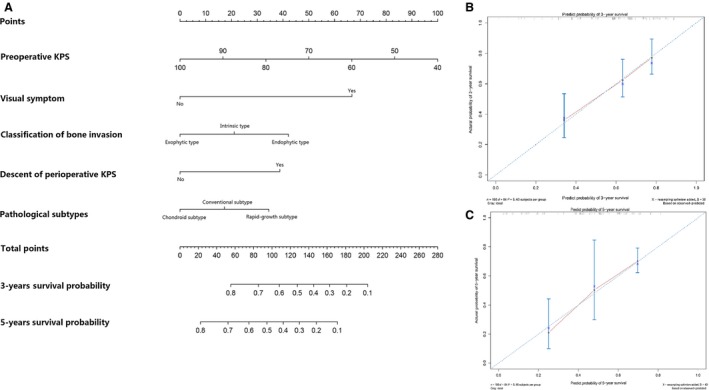
Nomogram for 3‐year and 5‐year PFS in patients with primary skull base chordoma (A) and calibration curve for internal validation of the 3‐year (B) and 5‐year (C) nomogram. To calculate the survival probability for a specific patient by (A), locate patient preoperative KPS score and draw a line straight upward to the Points axis to determine the score associated with that KPS score. Repeat the process for visual symptom, classification of bone invasion, descent of perioperative KPS, and pathological subtypes, sum the scores for each factor, and locate this sum on the Total Points axis. Then, draw a line straight down to the corresponding 3‐year and 5‐year survival probability to find the predicted PFS probability. In the calibration plots of (B) 3‐year and (C) 5‐year PFS, nomogram‐predicted probability of PFS is plotted on the *x*‐axis; actual PFS is plotted on the *y*‐axis. The dashed line is the reference line, indicating where an ideal nomogram would lie. The Xs represent observed survival corrected‐for‐optimism in the same subgroup. KPS, Karnofsky performance scale; PFS, progression‐free survival.

The nomogram based on the Cox proportional hazards model of primary group is shown in Figure 3A. Using the nomogram, 3‐year and 5‐year PFS probability can be estimated from individual patient and tumor characteristics. The prognostic factors in nomogram were in line with Cox multivariable analysis by SPSS aside from the addition of two parameters about KPS scores. Negative prognostic factors contributed fewer points so that increasing total points were associated with increasingly worse prognosis. A more detailed description of nomogram use was given in the Figure 3 legend. The nomogram was internally validated by the calibration plot in Figure 3B and C, and by computing the bootstrap‐corrected Harrell C statistic. The calibration plot suggested that the nomogram was well calibrated; predicted and observed survival were in good agreement (circles lying almost directly on the reference line), with only minor discrepancies between observed (circles) and corrected‐for‐optimism (Xs) survival. A relatively high C statistic (0.67) was obtained, indicating good model discriminative ability.

## Discussion

There were, as far as we known, only a few retrospective reports that distinguished primary skull base chordomas from recurrent ones 9, 10, 18. In this study, these two groups demonstrated various characteristics in aspects of clinicopathologic features and progression‐free survival time. It was necessary to distinguish primary cases from recurrent ones in prognostic analysis.

Our findings about tumor progression in this kind of rare neoplasm verified that this issue should be regarded as not only an inevitable result, but also a signal of accelerating progress. Firstly, as being coincident with previous studies 9, the mean pr‐PFS time was significantly shorter in recurrent group than primary group. Secondly, by the self‐contrast analysis, the obvious change between the initial PFS time and postrecurrent PFS time was found in the eight patients who underwent the events of tumor progression twice. If we look for reasonable explanations from their clinicopathologic correlations, a significantly higher ratio of rapid‐growth subtype in the recurrent cases seemed to come to the fore. Although rapid‐growth subtype, according to different pathological classification criteria, had been proved to confer significant PFS disadvantages 5, 12, 13, 14, 15, self‐contrast pathological analyses between primary and recurrent tumor tissues are necessary to get the exact answer. In addition, whether and how surgery, the only intervention factor in the eight cases, had an impact on the tumor's molecular biological characteristics, such as the promotion of malignancy level, was still uncertain, which is still in need of further studies.

Regarding factors for tumor progression, we found that marginal resection was a strong determinant of better PFS in both primary and recurrent groups, which was attempted to be achieved whenever possible, especially as patients' initial treatment choice 9, 10, 12.Though marginal resection, as an external factor, failed to be an independent prognostic factor according to the multivariable analysis of Group A, its importance in primary cases should not be overemphasized 19, 20, 21. Compared with some other series that consisted of comprehensive treatment protocols 7, the comparable long‐term outcome of this cohort mainly relied on the relatively high proportion of marginal resection, including radical resection which was combined with marginal resection in this study. The role of postoperative radiotherapy on primary cases' survival cannot be fairly judged by this study and needs further research. The only relatively reliable finding about adjuvant radiotherapy in this study, referring mainly to Gamma knife, is that it can bring more progression‐free survival benefit to the patients with recurrent lesions 22.

In this study, the clinical and radiological features rather than treatment modalities seemed to be more associated with progression of primary lesions. Age, when cutting‐off point was set as 30 years, did demonstrate a significant effect on PFS when regarding this cohort as a whole, but it failed to became a prognostic factor when primary and recurrent cases were analyzed separately. Although the controversies on whether age can gain prognostic relevance or not cannot be ended 6, 10, 12, 15, 18, 20, 21, 23, because only one potential cutt‐off point, 30 years, was tested in this study, this result highlighted the importance to distinguish recurrent from primary lesions in prognosis analysis once again.

The other three findings worthy to be mentioned were visual disturbance as initial symptoms, which was identified as an independent risk factor in both groups, perioperative KPS scores, and degree of bone invasion. Although visual deficits constituted a worse rate of tumor control, it was proposed for the first time, the possible reason caused by the disparity of sample size in this study cannot be excluded. Factors regarding function status, preoperative KPS ≤ 70, and declined postoperative KPS scores were proved as prognostic factors for PFS in primary patients, which were consistent with prior studies 24. On the other hand, the predictive value of perioperative KPS scores in recurrent patients was not as strong as in the primary group, and it may be caused by the increasing convergence of the data in Group B. The various degrees of bone invasion, which meant that the heavier the bone structures of skull base had been destroyed, the more residual would be left postoperatively, resulting in earlier recurrence, was proposed as a rational predictor of tumor progression in this study for the first time.

Another vital issue to be mentioned was the definitions of pathological subtypes in this study. Firstly, the pathological diagnosis of chondroid chordoma was made after chondrosarcoma was definitely excluded 3, 4. Secondly, the definition of dedifferentiated chordoma in *Atlas of Tumor Pathology* had been viewed as the main reference for the corresponding diagnosis in this series 25. Thirdly, the criteria of rapid‐growth subtype was newly proposed in this study based on a series of prior studies, in which necrosis and mitosis gained prognostic superiority to other features such as atypia, inflammation, and cellularity 5, 14, 15.

Our findings on the basis of pathological results can be summarized as: (1) compared with conventional subtype, chondroid subtype was an independent protective factor for progression in primary cases, which was supported by other previous reports 4, 6, 20, and a potential protective factor for pr‐PFS; (2) rapid‐growth subtype, which was labeled as tumoral necrosis, mitosis, and the cell marker Ki‐67 ≥ 6% in this series, exhibited an unusually fast rate of growth and acted as an adverse prognostic factor of PFS 12, 13, 14, 15, 16, 23, 24. As a result, the current histopathological classification of chordoma was proved to be insufficient to describe the heterogeneity which was strongly associated with patients' long‐term outcome. A more comprehensive histopathologic grading system^5^, ^10^, ^14^ or some more convincing molecular markers are of necessity 23, and by which chordoma can be subdivided as more subentities that differ from each other in terms of prognosis.

Because this series was relatively large with sufficient follow‐up information, and strong prognostic associations were found, we decided to develop a nomogram to predict 3‐year and 5‐year PFS. We proposed the nomogram as a useful predictor of tumor progression in individual patients and as a useful tool for risk stratification in clinical studies. It is noteworthy that the factors included in the nomogram were all related to the tumors' inherent features rather than acquired factors such as treatment strategies, and a much wider applicability of this nomogram may be guaranteed.

Nevertheless, there are some limitations of this study. Firstly, selection bias, which is inherent in retrospective studies, cannot be avoided, and it could bias the results in the way that our treatment philosophy implied. Secondly, because of the absence of external verification, utility of the nomogram for decision‐making in patients who receive different treatment strategies from ours, such as a cytoreductive surgery followed by proton‐beam radiotherapy, remained uncertain and needed to be confirmed.

To conclude, we found that factors for tumor progression demonstrated some difference between primary and recurrent cases. We also found that a tendency of accelerating progress existed after tumor recurrence. The nomogram of tumor progression, which was consisted by factors of initial symptom, biological behavior of bone invasion, pathological characteristics, and functional status, was well established for risk stratification of primary cases.

## Conflict of Interest

There is no conflict of interest for all authors in this study.
